# A Novel Physical Layer Assisted Authentication Scheme for Mobile Wireless Sensor Networks

**DOI:** 10.3390/s17020289

**Published:** 2017-02-04

**Authors:** Qiuhua Wang

**Affiliations:** School of Cyberspace, Hangzhou Dianzi University, Hangzhou 310018, China; wangqiuhua@hdu.edu.cn; Tel.: +86-571-8687-3820

**Keywords:** wireless sensor networks, physical layer authentication, channel reciprocity, spatial uncorrelation, hypothesis testing

## Abstract

Physical-layer authentication can address physical layer vulnerabilities and security threats in wireless sensor networks, and has been considered as an effective complementary enhancement to existing upper-layer authentication mechanisms. In this paper, to advance the existing research and improve the authentication performance, we propose a novel physical layer assisted authentication scheme for mobile wireless sensor networks. In our proposed scheme, we explore the reciprocity and spatial uncorrelation of the wireless channel to verify the identities of involved transmitting users and decide whether all data frames are from the same sender. In our proposed scheme, a new method is developed for the legitimate users to compare their received signal strength (RSS) records, which avoids the information from being disclosed to the adversary. Our proposed scheme can detect the spoofing attack even in a high dynamic environment. We evaluate our scheme through experiments under indoor and outdoor environments. Experiment results show that our proposed scheme is more efficient and achieves a higher detection rate as well as keeping a lower false alarm rate.

## 1. Introduction

As wireless sensor networks (WSNs) become increasingly popular, the security and reliability issues attract more and more attention. Due to the broadcasting and open nature of wireless mediums, WSNs are vulnerable to identity-based spoofing attacks, where an unauthorized user attempts to impersonate another legitimate user in order to gain illegitimate advantages. Identity-based attacks are very easy to launch and are considered as the first step for attackers launching various types of attacks such as session hijacking, denial of service (DOS) and man-in-the-middle [1]. For example, in commodity networks, such as 802.11 networks, it is easy for a device to alter its media access control (MAC) address and claim to be another one by simply using an “ifconfig” command. Hence, the ability to distinguish between different transmitters would be particularly valuable for preventing spoofing attacks.

Authentication is an effective approach to deal with such identity-based attacks, by which the intended receivers can verify the identities of involved transmitting users and make sure that the received data come from the expected user [2]. Traditional authentication mechanisms are handled at the upper-layer using key-based cryptography. Although traditional cryptographic techniques can potentially prevent identity-based attacks in WSNs, they are either inefficient or suffer from constraints in certain existing scenarios. First, the security key distribution and management over large-scale or dynamic WSNs is a big challenge, and may not even be feasible in highly dynamic network environments. Second, once these pre-distributed keys are compromised, the whole security of authentication mechanisms will be broken. Third, traditional authentication mechanisms are accomplished by relying solely on the upper layers of protocol stack. They often lead to excessive communication overheads, computational load, transmission latency and high power consumption, thus decreasing the network capacity [3]. Furthermore, traditional upper-layer authentication does not directly address physical layer vulnerabilities and security threats from the open air interface.

In order to address these weaknesses of traditional authentication techniques, a variety of physical-layer authentication schemes have been proposed to enhance security in wireless networks. These schemes utilize the physical channel properties such as channel state information (CSI) or received signal strength (RSS) as a new form of fingerprint to distinguish between a legitimate transmitter and an illegitimate one [4,5,6,7,8,9,10,11,12,13,14,15]. The foundation behind them is that the CSI and RSS are spatial and temporal uniqueness to the legitimate sender and receiver. An attacker who is at a different location from the legitimate users will experience independent fading and thus cannot measure the same CSI or RSS profiles as the legitimate users [16,17,18]. Based on this spatial uncorrelation principle, the receiver can distinguish between the legitimate sender and the spoofer. Specifically, in physical-layer authentication schemes, the receiver authenticates the identity of transmitters at different locations by continuously comparing current CSI or RSS with the previous legitimate one. To enable the receiver to obtain the first CSI or RSS of the legitimate channel, it is assumed that a training/probing sequence is transmitted and authenticated based on an upper-layer authentication scheme prior to the adversary’s arrival. Physical-layer algorithms are usually performed by integrating channel measurements with hypothesis testing to determine whether the new message comes from the source of prior one.

Compared with traditional key-based authentication mechanisms, wireless channel characteristics are very hard to mimic. Consequently, physical-layer authentication can achieve fast and efficient message authentication [2], and therefore has been recently used as a complementary enhancement to existing upper-layer authentication [3].

### 1.1. Related Works and Issues

In recent years, various efforts have been made in exploring physical layer characteristics as fingerprints to distinguish among different transmitters in wireless networks [4,5,6,7,8,9,10,11,12,13,14,15,19,20,21]. In these works, physical-layer authentication is performed by comparing a measured channel characteristic with a prior one. In [4,9], the authors proposed to integrate channel probing mechanisms with hypothesis testing to determine whether the current and prior messages are sent by the same user. In their schemes, they used the consecutive channel frequency response (CFR) as the channel information fingerprint. In [11,21], the channel impulse response (CIR) was used to identify the transmitter. Although CFR and CIR can provide accurate channel information, they are not available in current device drivers, which prevents their practical usage for commodity wireless devices. Instead, in [10,19,20], a coarser indicator of the channel information, RSS, is in-depth and is used as fingerprint to implement authentication. As RSS is easily available in the current wireless devices, it has been widely used to authenticate transmitters in the real systems.

Most of the current physical-layer authentication schemes require the legitimate user to be authenticated at a specific location, and thus are applicable only for static networks. They cannot work well in dynamic environments where the channel state information changes drastically over time due to fading or mobility. In mobile environments, these schemes typically generate excessive false alarms, and the performance degrades significantly [8]. The challenge of using channel properties to assist authentication in a mobile environments is mainly due to the fact that the channel profile tends to change over time due to the nodes’ mobility and changing environment. It is difficult to obtain a relatively stable channel profile for a mobile node. Hence, it is hard to decide whether the channel profile variation is caused by the node’s mobility or a spoofing attack [5].

Limited work has considered mobile scenarios. In [8], the authors proposed an intra-burst and inter-burst authentication framework by using CSI for mobile networks. The intra-burst authentication was performed by comparing the current CSI with the previous one. However, the concrete inter-burst authentication scheme is not given in [8]. In [5], the authors proposed an RSS based intra-burst and inter-burst authentication scheme. For the intra-burst authentication, the receiver compares the RSS of the *n*th DATA frame with that of the (*n* − 1)th DATA frame. If the difference is less than a certain threshold, the receiver assumes that the *n*th DATA frame is sent by the expected sender. Otherwise, he will generate an alarm. For inter-burst authentication, the sender sends the receiver his RSS records of acknowledgement (ACK) frames during their previous communication. If these records are similar to the receiver’s version, the receiver assumes the sender be legitimate. Otherwise, he generates an alarm.

However, in many physical layer based key generation mechanisms [22,23,24,25,26,27], these RSS records are used as a random source to generate shared secret keys, and the generated shared secret keys are then used as encryption keys or authentication keys for secure communication. Once the adversary gets the RSS records of the legitimate users, he can extract the secret keys, and no communication security can be guaranteed in these physical layer based key generation mechanisms. Hence, those RSS records should be highly private and must not be transmitted on the public channel.

Therefore, how to compare the RSS records between the legitimate sender and receiver without transmitting them is a challenge.

### 1.2. Our Motivations and Contributions

In this paper, we focus on the physical-layer assisted authentication in mobile environments and consider the “data burst” communication model as in [5,8]. Our aim is to deal with the aforementioned challenge, advance the existing research and improve the authentication performance. We propose a novel inter-burst physical-layer authentication scheme for mobile wireless networks. Our method makes use of the inherent reciprocity and spatial uncorrelation of wireless fading channel to decide whether all data frames are from the same sender. Compared with the previous schemes, the main advantages of our proposed scheme are summarized as follows:

(1) In our inter-burst authentication scheme, a new method is developed for the legitimate users to compare their RSS records while not requiring the sender to send his RSS values back to the receiver, which avoids the information disclose to the attacker. Hence, our proposed scheme is more secure than that in [5] and causes no loss of secrecy. Our scheme can be combined with other RSS-based physical-layer secret key generation schemes [22,23,24,25,26,27] to further enhance the security of wireless networks.
(2) Our proposed inter-burst authentication scheme can detect the spoofing attack even in high dynamic environment.
(3) Our proposed inter-burst authentication scheme is more efficient since it achieves higher detection rate while keeping lower false alarm rate. In our scheme, the detection rate can achieve 100% without false alarm both in indoor and outdoor environments. Moreover, our solution is lightweight in the sense that only one short message communication is needed. Our scheme can achieve fast and efficient message authentication with less communication and computation overheads.
(4) We conduct the performance analysis on the inter-burst authentication, and identify its applicability.
(5) We validate our approach through real-world experiments under indoor and outdoor environments. Experiment results backs our proposed scheme well. Our scheme is a practical one.

### 1.3. Organization of the Paper

The rest of this paper is organized as follows. Section 2 introduces the theoretical basis used in our proposed scheme. Section 3 provides the detailed description of our proposed physical-layer assisted authentication scheme. Section 4 presents the experiment results and performance analysis, and identifies the applicability of our proposed scheme. Finally, we conclude the paper in Section 5.

## 2. Theoretical Basis

Our scheme is based on channel reciprocity, spatial uncorrelation, and two theorems that capture properties of correlated random sequences.

### 2.1. Channel Reciprocity Principle

In typical wireless network environments, the wireless channel between two users, Alice and Bob, is reciprocal and varies randomly over time and space [17,18]. Alice and Bob can measure some wireless channel characteristics (e.g., RSS and CSI). The channel reciprocity principle indicates that bidirectional wireless channel states are identical between two transceivers at a given instant of time. We can use these measurements as shared random secrets to achieve the message authentication. Specifically, let $X_{A}=(x_{A1},x_{A2},...,x_{An})$ be the channel measurements recorded by Alice at time $t_{1},t_{2},...,t_{n}$, respectively, and $X_{B}=(x_{B1},x_{B1},...,x_{Bn})$ be the channel measurements recorded by Bob at time $t_{1^{\prime }},t_{2^{\prime }},...,t_{n^{\prime }}$, where $t_{1}<t_{1^{\prime }}<t_{2}<t_{2^{\prime }}<...<t_{n}<t_{n^{\prime }}$ and $x_{Ai}$ and $x_{Bi}$ are the channel measurements at time $t_{i}$ and $t_{i^{\prime }}$. According to the reciprocity of wireless channel, $x_{Ai}\approx x_{Bi}$ (1 ≤ *i* ≤ *n*), if they are obtained within the channel coherence time $t_{\tau }$, i.e., $t_{i^{\prime }}-t_{i}<<t_{\tau }$ [17,18].

Therefore, since $x_{Ai}\approx x_{Bi}$ (1 ≤ *i* ≤ *n*), the correlation coefficient of sequence $X_{A}$ and $X_{B}$ will be very close to 1. The definition of correlation coefficient is given in Equation (1)

(1) $$\rho_{AB}=\rho (X_{A},X_{B})=\frac{E\{[X_{A}-E(X_{A})][X_{B}-E(X_{B})]\}}{\sqrt{D(X_{A})}\sqrt{D(X_{B})}}$$

where $\rho (X_{A},X_{B})$ stands for the correlation coefficient of $X_{A}$ and $X_{B}$, and E(X) is the expectation of *X* and D(X) is the variance of *X*.

### 2.2. Spatial Uncorrelation Principle

The spatial uncorrelation principle indicates that the properties of wireless channel are unique to the locations of the two transceivers of the link. An eavesdropper at a third location more than one-half wavelength away from the legitimate transceivers experiences independent channel variations and measures different, uncorrelated wireless channel states [16,17,18]. Specifically, the adversary, Eve, can listen to all communication between Alice and Bob. Eve can measure the channel between herself and Alice or Bob, however, according to the spatial uncorrelation principle, if Eve is more than λ/2 (λ is the wavelength) away from Alice and Bob, her channel measurements $X_{AE}=(x_{AE1},x_{AE2},...,x_{AEn})$ and $X_{BE}=(x_{BE1},x_{BE2},...,x_{BEn})$ are sufficiently uncorrelated with $X_{A}=(x_{A1},x_{A2},...,x_{An})$ and $X_{B}=(x_{B1},x_{B1},...,x_{Bn})$. Hence, the correlation coefficient of $X_{AE}$ and $X_{A}$, $X_{BE}$ and $X_{B}$ will be close to 0. Using this principle, the receiver can distinguish between legitimate sender and the spoofer.

### 2.3. Two Theorems

**Theorem** **1.** *Suppose that* $X_{A}$ *and* ${X_{A}}^{\prime }$ *are two uncorrelated random sequences,* $E(X_{A})=\mu_{A}$*,* $E({X_{A}}^{\prime })={\mu_{A}}^{\prime }$*,* $D(X_{A})=D({X_{A}}^{\prime })=\sigma^{2}$ *and* $\mathrm{cov}(X_{A},{X_{A}}^{\prime })=0$*. Define a new random sequence* $Y=\rho X_{A}+\sqrt{1-\rho^{2}}{X_{A}}^{\prime }$*. Then, this new sequence* Y *has a correlation coefficient of* ρ *with sequence* $X_{A}$*, that is,* $\rho_{YX_{A}}=\rho (Y,X_{A})=\rho$.

**Proof.**  See Appendix A. □

**Theorem** **2.** *If* $\rho_{X_{A}X_{B}}=\rho (X_{A},X_{B})=1$*, then for random sequence* $Y=\rho X_{A}+\sqrt{1-\rho^{2}}{X_{A}}^{\prime }$*,* $\rho (X_{A},Y)=\rho (X_{B},Y)=\rho$.

**Proof.**  See Appendix B. □

## 3. Our Proposed Authentication Scheme

In this paper, we propose a novel inter-burst authentication scheme for mobile environments without requiring Alice to send her measured channel information back to Bob. As CSI is not available in current device drivers, we make use of the most popular channel characteristic parameter, RSS, as the indicator of the channel because its reading is readily available in existing wireless devices. Most of the current off-the-shelf wireless cards, without any modification, can measure it on a per packet/frame basis. RSS can be read during the preamble stage of the receiving frame. The variation over time of the RSS caused by motion and multipath fading can be used as fingerprints for authentication.

It should be noted that our approach is also applicable to any other parameters of channel characteristic, such as amplitude or phase, etc.

### 3.1. System and Communication Model

**Figure 1:**
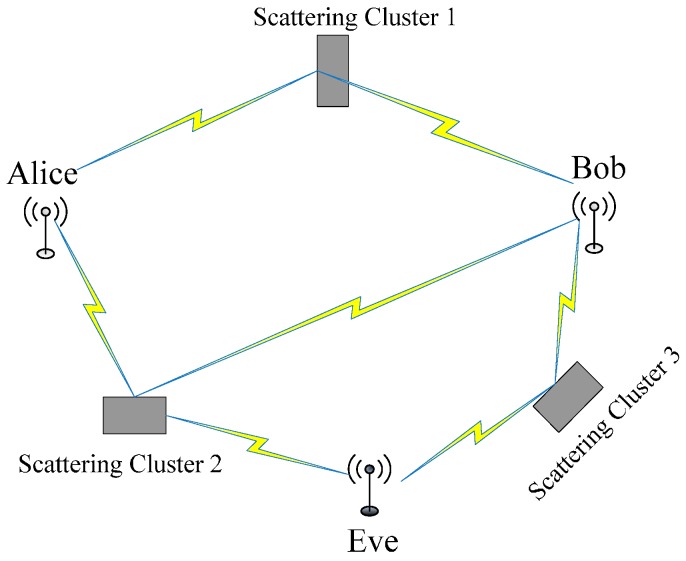
Wireless communication scenario.

Similar to most physical-layer authentication schemes, we consider an ubiqitous “Alice-Bob-Eve” wireless communication scenario in Figure 1, in which Alice, Bob and Eve are geographically located at different positions. The legitimate users, Alice and Bob, need to transmit messages via wireless channels. For the sake of discussion, we assume that Alice is the initiator, while Bob is the intended receiver. An active attacker, Eve, tries to inject messages into the medium in the hope of impersonating Alice by changing her MAC address into Alice’s. Eve can also passively overhear the messages transmitted between Alice and Bob. Bob should have the ability to detect Eve’s spoofing attack even when the packet sent from Eve carries the same identity as Alice’s.

In our setting, the bidirectional channel properties are provided by the DATA-ACK pair. Bob records the RSSs (represented by sequence $X_{B}$) of the DATA frames sent from Alice; Alice records the RSSs (represented by sequence $X_{A}$) of the corresponding ACKs sent back by Bob. For each DATA frame and ACK frame, Eve also records her RSSs (represented by sequences $X_{AE}$ and $X_{BE}$) even though they are not sent to her. Based on the above principles and theorems in Section 2, the correlation coefficient of $X_{A}$ and $X_{B}$ will be close to 1 (i.e., $\rho (X_{A},X_{B})\approx 1$), while the correlation coefficient of $X_{AE}$ and $X_{A}$ or $X_{BE}$ and $X_{B}$ will be close to 0 (i.e., $\rho (X_{AE},X_{A})\approx 0$ and $\rho (X_{BE},X_{B})\approx 0$). We validate these results in Section 4.1, and the experimental results are $\rho (X_{A},X_{B})=0.983$, $\rho (X_{AE},X_{A})\approx 0.093$ and $\rho (X_{BE},X_{B})\approx 0.106$ with signal-to-noise ratio (SNR) of 20 dB.

**Figure 2:**
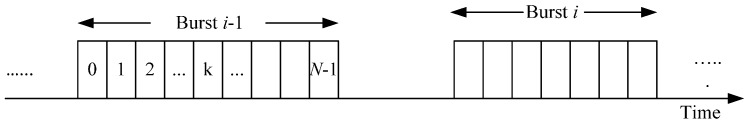
Communication model.

Similar to [5,8], we consider the following communication model in Figure 2. After Alice and Bob transmitted *N* frames consecutively, they may stop for a while before they start another consecutive transmission. We call the transmission of consecutive *N* frames as a “data burst”. The whole session between Alice and Bob may consist of several “data burst”, and the number of frames *N* in each burst may vary. The interval between two consecutive DATA frames in each burst is assumed to be much smaller than the channel correlation time. Thus, we perform intra-burst authentication within a “data burst”. However, the time interval between two consecutive “data bursts” may be much larger, (e.g., much larger than the channel coherence time, during which Alice may have moved a significant distance). Thus, the RSS of the first DATA frame in the current “data burst” may become totally uncorrelated with the RSS of the last DATA frame in the previous “data burst”, and the intra-burst authentication cannot work. Under this situation, we use the inter-burst authentication scheme to bridge the gap between bursts of communications.

### 3.2. Authentication Scheme

(1) Intra-burst authentication

The same as [5], the intra-burst authentication is used for per frame authentication within a “data burst”. It is based on the assumption that the channel state or RSS of consecutive DATA frames are similar during the channel coherence time.

Assume that Bob has authenticated the *n*th DATA frame coming from Alice and has recorded its RSS, *RSS*(*n*). After receiving the (*n* + 1)th DATA frame, Bob records its RSS, *RSS*(*n* + 1), and compares it with *RSS*(*n*). As the measurement time interval between the two consecutive DATA frames is less than the channel coherence time, if the two RSSs are “close” to each other (the difference is less than some threshold), Bob then concludes that the channel profile variation is caused by the node’s mobility, and he will assume that the current DATA frame is still from Alice. Otherwise, if the two RSSs are not similar (the difference exceeds some threshold), Bob then assumes that the channel profile variation is caused by the spoofing attack, that is, the current frame is likely sent by an attacker. More specifically, Bob uses a simple hypothesis test to decide whether the current (*n* + 1)th DATA frame still comes from Alice or a would-be attacker, e.g., Eve:

(2) $$H_{0}:|RSS(n+1)-RSS(n)|\leq \eta$$

(3) $$H_{1}:|RSS(n+1)-RSS(n)|>\eta$$

The null hypothesis, $H_{0}$, means that the current frame is still sent by the legitimate transmitter, Alice, while, the alternative hypothesis, $H_{1}$, means that the current frame is from the spoofer, Eve. η is a threshold of the decision making for authentication. If the difference is equal to or smaller than η, Bob accepts the hypothesis $H_{0}$ and assumes that the (*n* + 1)th frame comes from Alice. Otherwise, he accepts the hypothesis $H_{1}$ and generates an alarm.

(2) Inter-burst authentication

When the time interval between two consecutive “data bursts” is so large that Alice has moved a significant distance, the RSS of the received DATA may become totally uncorrelated with that of the previous one. For such situations, we proposed an inter-burst authentication scheme. Our scheme is based on the wireless channel reciprocity principle, the spatial uncorrelation theory, and Theorems 1 and 2.

Different from [5], in our proposed scheme, instead of sending Bob a list of RSS of ACK frames, Alice sends a random sequence, which discloses no information about Alice’s RSS. Hence, our proposed scheme is more secure than [5] and causes no loss of secrecy. Our scheme can combined with other RSS-based physical layer secret key generation schemes [22,23,24,25,26,27] to further enhance the security of wireless networks.

Suppose that, during the previous “data burst” transmission, Alice has recorded the RSS values of the corresponding ACKs sent from Bob, denoted as sequence $X_{A}$, $E(X_{A})=\mu_{A}$, $D(X_{A})=\sigma^{2}$. Bob has recorded the RSS values of the DATAs sent from Alice, denoted as sequence $X_{B}$. All the frames in the previous “data burst” have been already authenticated by the intra-burst authentication or other upper-layer authentication mechanisms. Our proposed scheme is described as follows:

(1) If the current frame is the last one in a “data burst”, Alice adds an ending-flag and sends it to Bob. Once detecting the ending-flag, Bob chooses a random parameter ρ and adds it to its ACK frame. Bob also records the RSS of the last frame from Alice.

(2) Once receiving the ACK frame containing parameter ρ, Alice records its RSS and computes a new sequence $Y=\rho X_{A}+\sqrt{1-\rho^{2}}{X_{A}}^{\prime }$. Then, Alice sends Y to Bob as an authentication frame. ${X_{A}}^{\prime }$ is a random sequence which is uncorrelated with $X_{A}$, $E({X_{A}}^{\prime })={\mu_{A}}^{\prime }$ and $D({X_{A}}^{\prime })=\sigma^{2}$. According to Theorem 1, sequence $X_{A}$ has a correlation coefficient of ρ with sequence Y, that is, $\rho_{X_{A}Y}=\rho (X_{A},Y)=\rho$.

(3) After receiving the authentication frame, Bob records its RSS. Then, using his recorded RSS sequence $X_{B}$, Bob computes the correlation coefficient $\rho (X_{B},Y)$. According to Theorem 2, correlation coefficient $\rho (X_{B},Y)$ will be very close to ρ if this frame is from Alice or no attack within the previous “data burst”. Bob uses a hypothesis test to decide whether the current authentication frame comes from Alice or Eve:

(4) $$H_{0}:\;\Lambda_{\rho }=|\rho (X_{B},Y)-\rho |\leq \rho_{th}$$

(5) $$H_{1}:\;\Lambda_{\rho }=|\rho (X_{B},Y)-\rho |>\rho_{th}$$

The null hypothesis, $H_{0}$, means that the current authentication frame is sent by Alice or no attack within the previous “data burst”, while the alternative hypothesis, $H_{1}$, means that the current frame is from Eve or there are attacks within the previous “data burst”. $\rho_{th}$ is a threshold of the decision making for authentication. If the difference is equal to or smaller than $\rho_{th}$, Bob accepts the hypothesis $H_{0}$ and sends Alice an ACK frame. Otherwise, he accepts the hypothesis $H_{1}$ and generates an alarm.

The threshold $\rho_{th}$ of $\Lambda_{\rho }$ has no closed-form expression and has to be determined by simulations, as we show in Section 4.3.

(4) After passing the inter-burst authentication, Alice starts to send her next “data burst”, and Bob uses intra-burst authentication to authenticate each frame within the “data burst”.

## 4. Performance Evaluations

The performance of the intra-burst authentication has been studied in various works [4,5,6,7,8,9,10,11,12,13,14,15]. In this section, we only evaluate the performance of our proposed inter-burst authentication scheme. We conducted our experiment on three sensor devices (acting as Alice, Bob and Eve) under an indoor environment (on the third floor of our lab building) and an outdoor environment (on the lawn of our school). The sensor device is equipped with CC2430 chip, and runs 802.15.4/ZigBee protocol in 2.4 GHz frequency band with 250 kbps of data rate. The transmission power is set to 1 mW and the modulation technique is Quadrature Phase Shift Keying (QPSK). Bob remains stationary while Alice moves randomly at a typical pedestrian velocity, about 1 m/s. Eve is configured to monitor mode and move randomly. Eve launches attack from a random location, and we evaluate the average case. Bob records the RSSs of DATA frames sent from Alice; Alice records the RSSs of the corresponding ACKs sent back by Bob. These DATA and ACK frames are all for data communication between Alice and Bob. For each DATA frame and ACK frame, Eve also records her RSSs even though they are not sent to her. The time interval between two consecutive “data bursts” is set to be 0.5 s.

### 4.1. Reciprocity and Spatial Uncorrelation Validation

**Figure 3:**
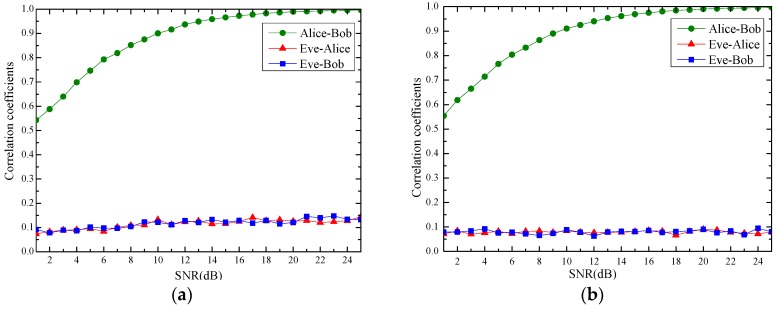
Reciprocity and spatial uncorrelation validation. (**a**) indoor environments; (**b**) outdoor environments.

In our experiment, Bob records the RSS values of DATA frames from Alice, and Alice records the RSS values of the corresponding ACK frames from Bob. We performed more than 100 times of the experiments, and the average correlation coefficients are shown in Figure 3.

Eve is able to capture and record the RSS values of all the DATA and ACK. The average correlation coefficients with Alice and Bob, respectively, are also shown in Figure 3.

From Figure 3, we can see that the correlation coefficient between Alice and Bob is very close to 1 (larger than 0.98 when SNR ≥ 18 dB), which justifies the reciprocity property. On the other hand, the RSS values observed by Eve are far from closely related to Alice’s or Bob’s. The correlation coefficient is less than 0.15 under the indoor case, and even less under the outdoor case, which verifies the spatial uncorrelation property. This experiment also demonstrates that the wireless fading channel state is a random secret shared by two transceivers.

### 4.2. Theorem 2 Validation

**Figure 4:**
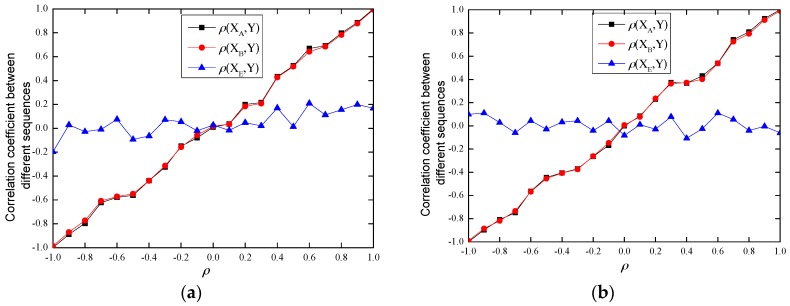
Theorem 2 validation. (**a**) indoor environments; (**b**) outdoor environments.

We validate Theorem 2 through experiments with ρ ranging from −1 to 1, and the results are shown in Figure 4. From Figure 4, we can see that the correlation coefficient between Alice’s sequence $X_{A}$ and the new generated sequence Y is very close to the correlation coefficient between Bob’s sequence $X_{B}$ and Y, i.e., $\rho (X_{A},Y)\approx \rho (X_{B},Y)$. We also show the correlation coefficient between Eve’s sequence $X_{E}$ and Y, and the results show that $\rho (X_{E},Y)$ is far different from ρ, $\rho (X_{A},Y)$ or $\rho (X_{B},Y)$, which further verifies the spatial uncorrelation property.

### 4.3. Performance Evaluation of Our Proposed Scheme

We evaluate the performance of our inter-burst authentication scheme by the false alarm rate *α* and the detection rate *β.* The false alarm rate is the probability of mistakenly declaring Alice as the attacker Eve when there is actually no attack. The detection rate is the probability of detecting the attacker when the attack really happens. Our goal is to achieve a high detection rate with a low false alarm rate.

For a given threshold $\rho_{th}$, the false alarm rate *α* and the detection rate *β* are defined, respectively, by,

(6) $$\alpha (\rho_{th})=P(\Lambda_{\rho }>\rho_{th}|H_{0})$$

(7) $$\beta (\rho_{th})=P(\Lambda_{\rho }>\rho_{th}|H_{1})$$

A good authentication scheme is one for which the false alarm *α* is low while the detection rate *β* is high. The performance of our inter-burst authentication scheme is related to parameter ρ and the test threshold $\rho_{th}$, which can be determined through experiments.

#### 4.3.1. The Choosing of Parameters ρ and $\rho_{th}$

**Figure 5:**
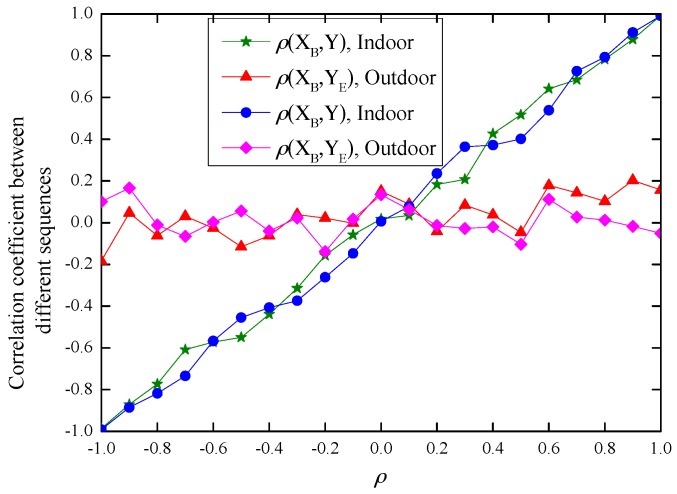
The experimental results of $\rho (X_{B},Y)$ and $\rho (X_{B},Y_{E})$.

The attacker Eve can eavesdrop the channel and measure the RSSs of the DATA and ACK frames transmitted between Alice and Bob, and she may also eavesdrop the random parameter ρ. Hence, Eve can generate the sequence $Y_{E}=\rho X_{E}+\sqrt{1-\rho^{2}}{X_{E}}^{\prime }$ based on her measured RSS sequence $X_{E}$ ($X_{E}$ may be $X_{AE}$ or $X_{BE}$) and parameter ρ using the same method as Alice. Then, Eve can send $Y_{E}$ to Bob hoping to pass the authentication. The experimental results of $\rho (X_{B},Y)$ and $\rho (X_{B},Y_{E})$ are shown in Figure 5.

**Figure 6:**
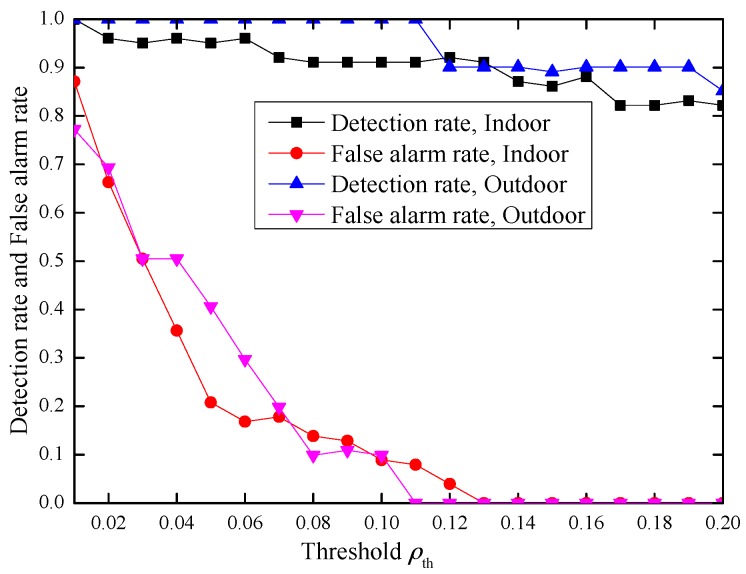
The average false alarm *α* and detection rate *β* with −1 ≤ ρ ≤ 1.

Theoretically, parameters ρ can range from −1 to 1, and the larger the threshold of $\rho_{th}$, the lower the detection rate and the false alarm rate. However, from experimental results in Figure 5, we notice that when ρ is small, (for example |ρ|<0.2 in our experiment), $\rho (X_{B},Y_{E})$ is close to $\rho (X_{B},Y)$. Under this case, when the threshold $\rho_{th}$ is small, the false alarm rate *α* will be high, while when the threshold $\rho_{th}$ is large, the detection rate *β* will decline. This observation is verified by experiment results in Figure 6.

From Figure 6, we can see that with ρ increasing, the difference between $\left|\rho (X_{B},Y)\right|$ and $\left|\rho (X_{B},Y_{E})\right|$ becomes large, and so does the difference between $\left|\rho (X_{B},Y)-\rho \right|$ and $\left|\rho (X_{B},Y_{E})-\rho \right|$. Hence, the performance of our scheme will improve. We verify this in the next Section 4.3.2.

#### 4.3.2. Performance Evaluation

**Figure 7:**
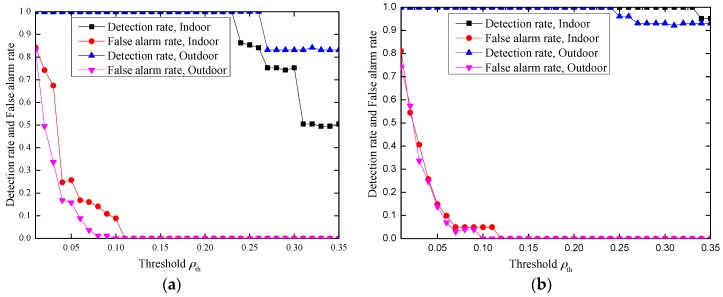
The average false alarm *α* and detection rate *β* with |ρ|>0.2 and |ρ|>0.3. (**a**) |ρ|>0.2; (**b**) |ρ|>0.3.

Based on the above analysis, in our experiment, we limit the range of parameter ρ to improve the performance. When *N* = 100 and the time interval between two consecutive “data bursts” is set to be 0.5 s, the experimental results are illustrated in Figure 7.

From the results, we can see that when |ρ|>0.2 and $0.11\leq \rho_{th}\leq 0.23$, the detection rate *β* can achieve 100% with false alarm rate *α* = 0 both in indoor and outdoor environments. Hence, our proposed inter-burst authentication scheme achieves effective message authentication and is a practical one.

#### 4.3.3. Impact of the Frame Number in Each Burst

**Figure 8:**
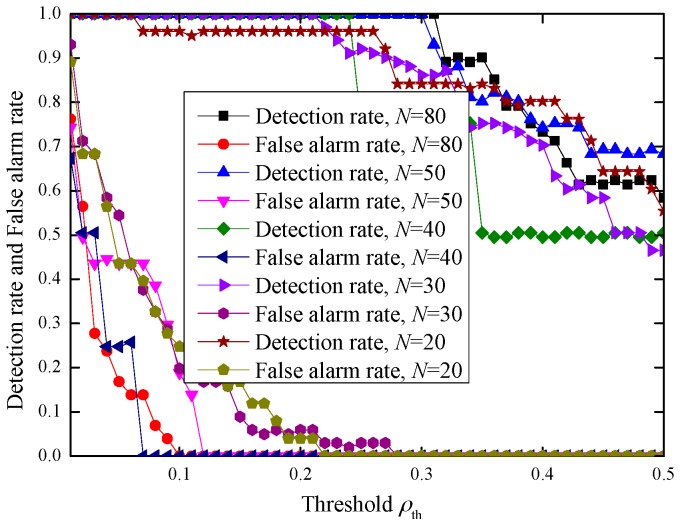
The impact of the frame number in an indoor environment, |ρ|>0.3.

The frame number in each burst is also a parameter that affects the performance. We simulate the impact of the frame number under an indoor environment with |ρ|>0.3. The experimental results are illustrated in Figure 8. From Figure 8, we can see that the detection performance improves with increased frame number. This may be due to the fact that the longer length results in a more accurate estimation of the correlation. In our experiment, a frame number of 40 can achieve 100% detection rate with no false alarm rate. Even when the frame number is 20, our scheme can still achieve 96% detection rate without introducing a false alarm.

**Figure 9:**
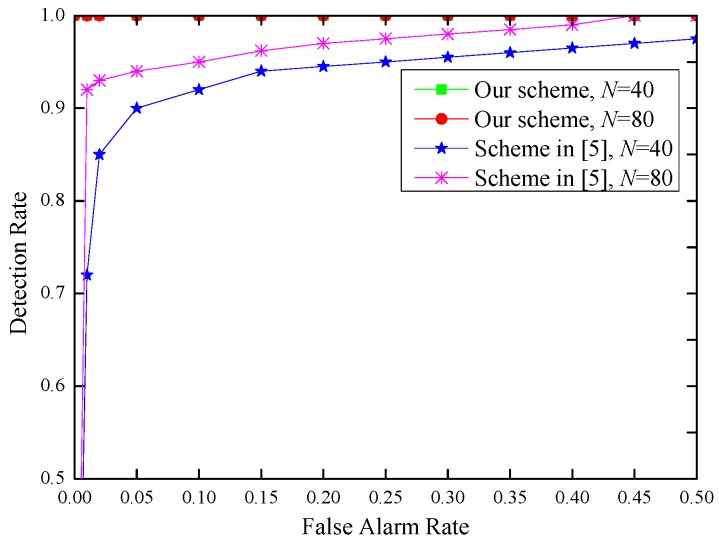
False alarm rate vs. detection rate in indoor environments, *N* = 40 and *N* = 80.

We further compare the performance of our proposed scheme with that in [5] with the frame number *N* = 40 and *N* = 80, respectively, and the results are shown in Figure 9.

From Figure 9, we can see that our proposed inter-burst authentication scheme is more efficient than the scheme in [5], since it achieves higher detection rate under the same conditions.

#### 4.3.4. Security

We consider that the attacker Eve can perform eavesdropping attacks and replay attacks.

In the eavesdropping attack, Eve eavesdrops the channel, and measures the RSS of the DATA and ACK frames transmitted between Alice and Bob, so Eve can generate the sequence $Y_{E}=\rho X_{E}+\sqrt{1-\rho^{2}}{X_{E}}^{\prime }$ based on her measured RSS sequence $X_{E}$ and parameter ρ using the same method as Alice, and send $Y_{E}$ to Bob hoping to pass the authentication. From the experimental results in Figure 5, when ρ is small while $\rho_{th}$ is large, the detection rate *β* will decline. However, from Figure 7, when |ρ|>0.2 and $0.11\leq \rho_{th}\leq 0.23$, our scheme can detect the attack with 100% detection rate. Hence, our scheme can prevent Eve’s eavesdropping attack.

In the replay attack, Eve replays Alice’s frame to Bob. However, since a fresh nonce is used for each frame, it is impossible for Eve to pass the authentication by replaying Alice’s frame.

In addition, different from [5], in our scheme, Alice sends Bob a random sequence instead of sending the real RSS values, which disclose no information about Alice’s RSS to Eve. Our scheme can be combined with other RSS-based physical-layer secret key generation schemes [22,23,24,25,26,27] to further enhance the security of wireless networks.

Hence, our proposed scheme is more secure than [5] and causes no loss of secrecy.

#### 4.3.5. Overheads

In our inter-burst authentication scheme, Alice sends a random sequence to Bob, which is considered as communication overhead. However, this communication overheard is very small. Assume that each burst has the same number of frames, *N*, and each value in the random sequence is represented by a byte. Then, Alice needs to transmit at most *N* bytes. For example, if the frame number in each burst is 100, Alice needs to send Bob at most 100 bytes, which can be included in one Zigbee packet (a Zigbee packet can carry at most 114 bytes payload). In fact, according to the experiment results in Figure 8, when |ρ|>0.3 and $0.07\leq \rho_{th}\leq 0.23$, a frame number of 40 already achieves a 100% detection rate with no false alarm rate. Even when the frame number is 20, our scheme can still achieve 96% detection rate without introducing false alarm.

For storage, our scheme requires both the sender and receiver to cache a RSS sequence. Hence, the storage overhead is also *N* bytes.

#### 4.3.6. Applicability

(1) Intra-burst authentication

The intra-burst authentication is used to authenticate each frame within a “data burst”. It is based on the assumption that the channel state or RSS of consecutive DATA frames are highly correlated during the channel coherence time. As the intra-burst authentication mechanism is the same as the current physical-layer authentication schemes, it is effective in static networks or relatively low speed networks, while its application is limited in high mobile environments. More specifically, in a wireless network with 2.4 GH carrier frequency, when the sender and receiver have low relative speed, e.g., at a walking speed of 1 m/s, the channel coherent time is $T_{C}=\frac{9c}{16\pi vf}=\frac{9\times 3\times 10^{8}}{16\pi \times 1\times 2.4\times 10^{9}}=22.4\;\mathrm{ms}$. In our WSN experiment with the data rate of 250 kbps and packet size of 127 bytes, the time difference between the measurements of two consecutive DATA frames is about 10 ms. Under this case, the intra-burst authentication is effective. However, with the moving speed increasing, the channel coherence time shortens. The performance degrades since it leads to smaller correlation between the channel measurements of two consecutive DATA frames. For example, in the highly mobile environment with a driving speed of 10 m/s, the channel coherence time is only 2.24 ms. Under this case, the measurement time interval between two consecutive DATA frames is larger than the channel coherence time. Hence, the intra-burst authentication will not work well in a highly mobile environment.

(2) Inter-burst authentication

Differing from the intra-burst authentication, the inter-burst authentication is based on the channel reciprocity theory that the channel state or RSS of the DATA frame and its corresponding ACK frame are highly correlated during the channel coherence time. As the measurement time difference between the DATA-ACK frame pair is smaller than the measurement time interval between the two consecutive DATA frames, the inter-burst authentication can still work well in a high dynamic scenario. In our experiments on WSNs, the measurement time difference between the DATA-ACK frame pair is about 4.5 ms with the data rate of 250 kbps and packet size of 127 bytes. This time difference is much smaller than the channel coherence time with the speed of 1 m/s. Therefore, the inter-burst authentication can work well in a pedestrian speed dynamic scenario. If the sensor nodes move at the bicycle speed of 4 m/s, the channel coherent time is 5.6 ms, and our inter-burst authentication can also work well. If the moving speed of the sensor nodes is faster than 4 m/s, the inter-burst authentication may not work. The main reason is that the data rate and the hardware processing speed of WSNs is much lower.

However, for the 802.11 wireless networks, the time difference between two measurements of the DATA-ACK frame pair is only about 0.5 ms with the data rate of 12 Mbps and packet size of 512 bytes, which is much smaller than the channel coherence time even in the vehicle speed environment. Hence, our inter-burst authentication can still work well in a high dynamic driving speed scenario.

Moreover, in the “data burst” communication scenario described in Section 3.1, when the time interval between two consecutive “data bursts” is much larger than the channel coherence time, or Alice has moved a significant distance between two consecutive “data bursts”, the RSS of the first DATA frame in the current “data burst” may become totally uncorrelated with the RSS of the last DATA frame in the previous “data burst”. Thus, the intra-burst authentication also cannot work. Under this situation, an inter-burst authentication scheme can be used to bridge the authentication gap between bursts of communications.

Therefore, in the low dynamic scenario, such as a pedestrian speed dynamic scenario, we can use the intra-burst authentication for per frame authentication within a “data burst”, and use the inter-burst authentication to bridge the authentication gap between bursts of communications. In the high dynamic scenario with bicycle speed and vehicle speed, although we cannot use intra-burst authentication to perform per frame authentication, we can still use the inter-burst authentication to detect whether there are spoofing attacks within the previous consecutive *N* frames. If all the consecutive *N* frames come from the real sender, the RSSs recorded by the sender should be correlated with the receiver’s recorders. Otherwise, the correlation will be degraded, and attacks can be detected. More specifically, assuming that the receiver, Bob, received *N* DATA frames from the same MAC address, and recorded each frame’s RSS as sequence $X_{B}$, the *N* DATA frames might be all sent by the real sender, Alice, or some of them are sent by the attacker, Eve. Bob then sends a verification request with parameter ρ to Alice for the consecutive *N* frames during their past communication. Upon receiving the verification request, Alice sends a new random sequence $Y=\rho X_{A}+\sqrt{1-\rho^{2}}{X_{A}}^{\prime }$ to Bob as described in Section 3.2. After receiving Y, Bob computes the correlation coefficient $\rho (X_{B},Y)$ and then uses the hypothesis test in Equations (4) and (5) to decide whether there is a spoofing attack within the consecutive *N* frames. In this case, the null hypothesis, $H_{0}$, means that all of the previous consecutive *N* frames are sent by Alice, that is, no spoofing attack happens, while the alternative hypothesis, $H_{1}$, means that some of them are sent by the attacker, that is, there is a spoofing attack within the consecutive *N* frames.

Note that the physical-layer authentication is mainly used to enhance or augment the existing upper-layer cryptography-based authentication mechanisms. For example, when the authentication key used by upper-layer authentication mechanisms is compromised, intra-burst and inter-burst authentication can be used as a complementary authentication method to verify the sender’s identity. Furthermore, traditional authentication mechanisms, such as HMAC (Hash-base Message Authentication Code), will tag an authentication code to each frame. However, the intra-burst authentication introduces no communication overhead. It only requires the receiver to cache one byte RSS value of the previous DATA frame. Our inter-burst authentication may introduce *N*-byte communication overhead. However, the introduced communication overhead is no more than traditional authentication mechanisms. For example, our inter-burst authentication needs to append a 20-byte random sequence to achieve desirable authentication strength as illustrated in Figure 8. HMAC, however, also needs to append a 20-byte authentication code if SHA-1 (secure hash algorithm) is adopted.

Since intra-burst authentication and inter-burst authentication only use simple comparison calculation, they are more computationally efficient than HMAC. As for storage, our inter-burst authentication requires both the sender and receiver to cache *N*-byte RSS sequences. However, the introduced storage overhead is less than traditional cryptographic mechanisms if 256-bit key is used.

## 5. Conclusions

In this paper, we focus on the issue of using the physical-layer authentication to enhance the existing upper-layer authentication mechanisms in mobile wireless sensor networks. We propose a novel physical-layer assisted inter-burst authentication scheme for mobile wireless sensor networks. Our proposed scheme is more secure since it discloses no information to the adversary. It can detect the spoofing attack even in a highly dynamic environment. We evaluate the proposed scheme through experiments under indoor and outdoor environments. The experimental results show that our proposed scheme can work reliably with high efficiency and that it achieves a higher detection rate and lower false alarm rate. Moreover, our scheme is practicable, light-weight and easily-implementable on the current wireless sensor devices.

## Appendix A

**Proof of Theorem 1.** Since $Y=\rho X_{A}+\sqrt{1-\rho^{2}}{X_{A}}^{\prime }$, $E(X_{A})=\mu_{A}$, $E({X_{A}}^{\prime })={\mu_{A}}^{\prime }$, $D(X_{A})=D({X_{A}}^{\prime })=\sigma^{2}$, then

(A1) $$\begin{array}{ll} E(Y) & =E(\rho X_{A}+\sqrt{1-\rho^{2}}{X_{A}}^{\prime })\\ & =\rho E(X_{A})+\sqrt{1-\rho^{2}}E({X_{A}}^{\prime })\\ & =\rho \mu_{A}+\sqrt{1-\rho^{2}}{\mu_{A}}^{\prime } \end{array}$$

(A2) $$\begin{array}{ll} E(Y^{2}) & =E[{\left(\rho X_{1}+\sqrt{1-\rho^{2}}X_{2}\right)}^{2}]\\ & =\rho^{2}E({X_{1}}^{2})+2\rho \sqrt{1-\rho^{2}}E(X_{1})E(X_{2})+(1-\rho^{2})E({X_{2}}^{2})\\ & =\rho^{2}{\mu_{A}}^{2}+\delta^{2}+2\rho \sqrt{1-\rho^{2}}\mu_{A}{\mu_{A}}^{\prime }+(1-\rho^{2}){{\mu_{A}}^{\prime }}^{2} \end{array}$$

(A3) $$\begin{array}{ll} D(Y) & =E\{{\left[Y-E(Y)\right]}^{2}\}\\ & =E(Y^{2})-{\left[E(Y)\right]}^{2}\\ & =\rho^{2}{\mu_{A}}^{2}+\delta^{2}+2\rho \sqrt{1-\rho^{2}}\mu_{A}{\mu_{A}}^{\prime }+(1-\rho^{2}){{\mu_{A}}^{\prime }}^{2}-{\left(\rho \mu_{A}+\sqrt{1-\rho^{2}}{\mu_{A}}^{\prime }\right)}^{2}\\ & =\delta^{2} \end{array}$$

Hence,

(A4) $$\begin{array}{ll} \rho_{YX_{A}} & =\rho (Y,X_{A})\\ & =\frac{E((Y-E(Y))(X_{A}-E(X_{A}))}{\sqrt{D(Y)}\sqrt{D(X_{A})}}\\ & =\frac{E(YX_{A})-E(Y)E(X_{A})}{\sqrt{D(Y)}\sqrt{D(X_{A})}}\\ & =\frac{E((\rho X_{A}+\sqrt{1-\rho^{2}}{X_{A}}^{\prime })X_{A})-(\rho \mu_{A}+\sqrt{1-\rho^{2}}{\mu_{A}}^{\prime })\mu_{A}}{\sigma^{2}}\\ & =\frac{\rho E({X_{A}}^{2})+\sqrt{1-\rho^{2}}E(X_{A})E({X_{A}}^{\prime })-\rho {\mu_{A}}^{2}-\sqrt{1-\rho^{2}}\mu_{A}{\mu_{A}}^{\prime }}{\sigma^{2}}\\ & =\frac{\rho ({\mu_{A}}^{2}+\sigma^{2})+\sqrt{1-\rho^{2}}\mu_{A}{\mu_{A}}^{\prime }-\rho {\mu_{A}}^{2}-\sqrt{1-\rho^{2}}\mu_{A}{\mu_{A}}^{\prime }}{\sigma^{2}}\\ & =\rho \end{array}$$

## Appendix B

**Proof of Theorem 2.** Since $\rho_{X_{A}X_{B}}=\rho (X_{A},X_{B})=1$, there exist constant numbers *a* and *b* that makes $P(X_{B}=aX_{A}+b)=1$. Then

(A5) $$E(X_{B})=E(aX_{A}+b)=a\mu_{A}+b$$

(A6) $$\begin{array}{ll} E({X_{B}}^{2}) & =E[{\left(aX_{A}+b\right)}^{2}]\\ & =E[a^{2}{X_{A}}^{2}+2abX_{A}+b^{2}]\\ & =a^{2}(\delta^{2}+{\mu_{A}}^{2})+2ab\mu_{A}+b^{2} \end{array}$$

(A7) $$\begin{array}{ll} D(X_{B}) & =E\{{\left[X_{B}-E(X_{B})\right]}^{2}\}\\ & =E({X_{B}}^{2})-{\left[E(X_{B})\right]}^{2}\\ & =a^{2}(\delta^{2}+{\mu_{A}}^{2})+2ab\mu_{A}+b^{2}-{\left(a\mu_{A}+b\right)}^{2}\\ & =a^{2}\delta^{2} \end{array}$$

Hence,

(A8) $$\begin{array}{ll} \rho_{X_{B}Y} & =\rho (X_{B},Y)\\ & =\frac{E[(X_{B}-E(X_{B})(Y-E(Y))]}{\sqrt{D(X_{B})}\sqrt{D(Y)}}\\ & =\frac{E(X_{B}Y)-E(X_{B})E(Y)}{\sqrt{D(X_{B})}\sqrt{D(Y)}}\\ & =\frac{E[(aX_{A}+b)Y]-E((aX_{A}+b))E(Y)}{a\sqrt{D(X_{A})}\sqrt{D(Y)}}\\ & =\frac{aE(X_{A}Y)+bE(Y)-aE(X_{A})E(Y)-bE(Y)}{a\sqrt{D(X_{A})}\sqrt{D(Y)}}\\ & =\frac{E(X_{A}Y)-E(X_{A})E(Y)}{\sqrt{D(X_{A})}\sqrt{D(Y)}}\\ & =\rho (X_{A},Y)\\ & =\rho \end{array}$$
